# Experimental Branch Retinal Vein Occlusion Induces Upstream Pericyte Loss and Vascular Destabilization

**DOI:** 10.1371/journal.pone.0132644

**Published:** 2015-07-24

**Authors:** Elisa Dominguez, William Raoul, Bertrand Calippe, José-Alain Sahel, Xavier Guillonneau, Michel Paques, Florian Sennlaub

**Affiliations:** 1 INSERM, U968, Paris, F-75012, France; 2 Sorbonne Universités, UPMC Univ Paris 06, UMR_S 968, Institut de la Vision, Paris, F-75012, France; 3 CNRS, UMR_7210, Paris, F-75012, France; 4 Université François-Rabelais de Tours, CNRS, GICC UMR 7292, Tours, France; 5 Centre Hospitalier National d¹Ophtalmologie des Quinze-Vingts, DHU ViewMaintain, INSERM-DHOS CIC 1423, Paris, F-75012, France; University of Florida, UNITED STATES

## Abstract

**Aims:**

Branch retinal vein occlusion (BRVO) leads to extensive vascular remodeling and is important cause of visual impairment. Although the vascular morphological changes following experimental vein occlusion have been described in a variety of models using angiography, the underlying cellular events are ill defined.

**Methods and Results:**

We here show that laser-induced experimental BRVO in mice leads to a wave of TUNEL-positive endothelial cell (EC) apoptosis in the upstream vascular network associated with a transient edema and hemorrhages. Subsequently, we observe an induction of EC proliferation within the dilated vein and capillaries, detected by EdU incorporation, and the edema resolves. However, the pericytes of the upstream capillaries are severely reduced, which was associated with continuing EC apoptosis and proliferation. The vascular remodeling was associated with increased expression of TGFβ, TSP-1, but also FGF2 expression. Exposure of the experimental animals to hypoxia, when pericyte (PC) dropout had occurred, led to a dramatic increase in endothelial cell proliferation, confirming the vascular instability induced by the experimental BRVO.

**Conclusion:**

Experimental BRVO leads to acute endothelial cells apoptosis and increased permeability. Subsequently the upstream vascular network remains destabilized, characterized by pericyte dropout, un-physiologically high endothelial cells turnover and sensitivity to hypoxia. These early changes might pave the way for capillary loss and subsequent chronic ischemia and edema that characterize the late stage disease.

## Introduction

Retinal vein occlusion (RVO) is the second most common retinal vascular disorder after diabetic retinopathy and is an important cause of visual impairment [1,2]. RVO can be classified in two types: central retinal vein occlusion (CRVO) that affects the entire retina, and branch retinal vein occlusion (BRVO) when any of the branches of the central vein is occluded. BRVO, the most common subtype of RVO, occurs in 0.6% to 1.1% in the population and only the retinal area dependent on the occluded vein is affected [3]. In humans, branch retinal veins do not directly connect with their neighboring veins. When a vein occludes the blood flow immediately stagnates and the pressure increases. As the blood from the dependent capillary bed can no longer be evacuated, the capillary blood flow slows down and the capillary pressure increases until it is drained through the capillaries and veins of the neighboring quadrants. The occluded vein dilates, becomes tortuous and hemorrhages appear as blood extravastes through the leaky vein and capillaries. The insufficiently irrigated retina becomes acutely edematous and vision in the affected retinal quadrant becomes blurry [4,5]. If the vein does not spontaneously recanalize, collateral veins develop that drain the blood from the affected quadrant through a neighboring vein [6],[7]. Subsequently, extensive capillary dropout can occur, leading to ischemia, chronic retinal edema and secondary neovascularization.

The acute events that follow the occlusion and lead to the immediate vascular remodeling and the predisposition to subsequent capillary dropout are ill defined. Venous blockage can be experimentally induced by laser photocoagulation and shares some significant similarities with the acute human disease [8–12]. Previous studies in rodent models of BRVO have described vascular remodeling using angiographies [11,12] and reported apoptosis in ganglion cell layer (GCL) and inner nuclear layer (INL) [8,13]. However, the cellular events that are at the origin of the vascular remodeling have never been described. We here investigated the acute vascular remodeling associated with retinal edema after experimental BRVO. We show that the acute retinal edema observed in experimental BRVO is associated with a wave of endothelial cell (EC) apoptosis. Subsequently, EC proliferate and the edema resolves. However, the capillaries in the affected sector remain dilated, which is associated with a durable loss of NG2^+^ pericytes (PC) and continued increase of EC apoptosis and proliferation. These cellular changes were associated with increased expression of TGFβ, TSP-1, but also FGF2 expression. Finally, we demonstrate that the vascular bed upstream of the occlusion had become destabilized, as it had become sensitive to hypoxia.

## Material and Methods

### Animals

Eight to 12 weeks-old C57BL/6JRj male mice were purchased from Janvier SA (Le Genest-Saint-Isle, France). Mice were maintained at the Institut de la Vision animal facility under pathogen-free conditions. All animals were housed in a 12h/12h light/dark cycle with food and water available ad libitum.

### Ethics statement

All experiments were performed after evaluation and approval of the Institutional Animal Care and Use Committee, Comité d'éthique pour l’expérimentation animale Charles Darwin (ID Ce5/2012/013), in accordance with the guidelines from Directive 2010/63/EU of the European Parliament on the protection of animals used for scientific purposes. All procedures were performed under anesthesia and all efforts were made to minimize suffering.

### BRVO model

Occlusion of one branch of the retinal vein was performed by laser photocoagulation as previously described [11,12] with slight modifications. Briefly, the pupils were dilated with tropicamide (Mydriaticum, Théa, France) and phenylephrin (Neosynephrine, Europhta, France).The animals were injected with 100μl of 1% fluorescein (Fluorescéine Sodique Faure 10%, Novartis Pharma, France) for dye enhancement and were immediately anesthetized by inhalation of isoflurane (Axience, France). The fundus was visualized with a slit lamp (BQ 900, Hagg-Streitt, Swiss) through a fundus laser lens (OFA 2.0 mm, Ocular Instruments Inc., USA) positioned on the mouse cornea with eye gel (Lubrithal, DechraVetxx, France). Seven to 12 laser impacts (0.5s, 200mW, 50μm spot size, Laser Yag 532 Eyelite (Alcon, USA)) were performed on a superior vein, 2 to 3 disc diameters (approximately 400–500μm) from the optic disc center. Sham procedure were performed with the same laser parameters necessary for the occlusion, but the laser was applied to a retinal area in between major vessels at the same distance from the optic nerve. Persistence of the occlusion was confirmed *in vivo* before sacrifice by intraperitoneal injection of fluorescein and fundus examination using Micron III device (Phoenix labs). Eyes with partial or complete recanalization observed in the angiography were excluded from the study.

### Spectral Domain-Optical Coherence Tomography (SD-OCT) imaging

Before and at 1, 3 and 7d after laser photocoagulation, retina of BRVO mice were visualized by a non-invasive exam with SD-OCT. After pupil dilatation, animals were anesthetized with isoflurane and placed in front of the SD-OCT imaging device (Bioptigen 840 nm HHP; Bioptigen, North Carolina, USA). Images were acquired from optic disc to approximately 1.4 mm of the superior retina including occlusion. Retinal layer thicknesses were measured at approximately 800–1000μm from the centre of the optic nerve, wich corresponds to an approximate distance of 400–500μm to the occlusion. SD-OCT was calibrated (1 pixel = 1.6 μm) and allowed reproducible measurements of retinal layer thickness at defined distance from the optic nerve. SD-OCT procedure and image calibration were performed as we previously described [14].

### Hypoxia model

Three days after BRVO, the persistence of the occlusion was checked by fundus visualization (MicronIII, Phoenix Laboratories, USA). A first group of animals (hypoxic group) were placed in Plexiglass chamber (ProOx P110 Oxygen Controller, Biospherix, USA) from d4 to d7 with free access to food and water. The level of oxygen was lowered to 10% inside the chamber through the infusion of nitrogen controlled by a feedback device (E702 Oxygen Sensor, Biospherix, USA). The second group (normoxic group) continued to be housed under normal animal housing conditions. Two supplementary groups with no BRVO were placed in normoxic or hypoxic conditions. All mice were daily injected with EdU from d3 to d7 before sacrifice.

### Immunohistochemistry

Mice were killed by CO_2_ inhalation and eyes were enucleated and fixed in 4% paraformaldehyde for 30 min at room temperature. After several washes in PBS, the cornea and lens were removed and the retina was carefully detached from RPE/choroid/sclera. The retinas were incubated overnight with primary antibodies (goat anti-collagen IV, 1:100, Serotec, USA; rabbit anti-NG2, 1:200, Millipore, USA) in PBS supplemented with 0.5% Triton X-100, followed by incubation with appropriate Alexa-coupled secondary antibodies (Life Technologies, France). Retinas were flatmounted and viewed with a fluorescence microscope (DM5500B, Leica, France) or with a confocal microscope (FV1000, Olympus, France). NG2+ pericytes nuclei were counted in occluded retina. Vascular areas (mm² occupied by vessels in the retina) were calculated with the “angiogenesis tube formation” add-in[15] available on MetaMorph 2D software (Molecular Devices, France). All cells counting were focused on ganglion cell layer of the retina.

### Terminal deoxynucleotidyl transferase dUTP nick end labeling (TUNEL)

After immunohistochemistry, retinal flatmounts were pre-treated with frozen methanol for 20 min and then frozen methanol/acetic acid (2:1) for another 20 min. After washing with PBS, the flatmounts were incubated overnight at 4°C with the reaction mixture as described by manufacturer’s protocol (In Situ Cell Death Detection Kit, Roche Diagnostics, USA) and then for 90 min at 37°C. After reaction was stopped by washing with PBS at RT, nuclei were counterstained with Hoechst (Sigma-Aldrich, France). The retinas were mounted, viewed and photographed with a fluorescence microscope (DM5500B, Leica) or with a confocal microscope (FV1000, Olympus).

### Cell proliferation

For in vivo cell proliferation analysis the mice were daily injected intraperitoneally with 5-ethynyl-2’-deoxyuridine (EdU, 50 mg/kg, Life Technologies) at d0 just after laser photocoagulation until sacrifice. The eyes were enucleated at d1, d3 or d7 and fixed in 4% paraformaldehyde for 30 min at room temperature and sectioned at the limbus; the cornea and lens were discarded. Retinas were dissected and incubated overnight with collagen IV antibody in 0.5% PBS Triton X-100 and revealed with appropriate secondary antibody. Detection of EdU was performed after immunohistochemistry with Click-IT EdU Imaging Kit (Life Technologies). The retinas were mounted, viewed and photographed with a fluorescence microscope (DM5500B, Leica) or with a confocal microscope (FV1000, Olympus).

### RT-PCR

At different time points after BRVO, the third of the retina containing the occluded vein (visible upon dissection) were carefully dissected in RNase-free conditions. Total RNA was isolated with Nucleospin RNAII (Macherey Nagel, France). Single-stranded cDNA was synthesized from total RNA (pretreated with DNaseI amplification grade, Life Technologies, France) using oligo-dT as primer and superscript II reverse transcriptase (Life technologies, France). Subsequent real-time polymerase chain reaction (RT-PCR) was performed using cDNA and SYBR green Gene Expression Master Mix (Life technologies) and primers (0.5 pmol/μl) available upon request. Results were normalized by expression of S26. PCR reactions were performed in 45 cycles of 15 s at 95°C, 45 s at 60°C.

### Statistical analysis

GraphPad Prism 5 (GraphPad Software, San Diego, USA) was used for data analysis and graphic representation. All values are reported as mean ± SEM. Mann-Whitney non parametric test was used for statistical analysis compared to control P < 0.05 was considered statistically significant.

## Results

### Experimental BRVO leads to acute inner retinal edema

**Fig 1:**
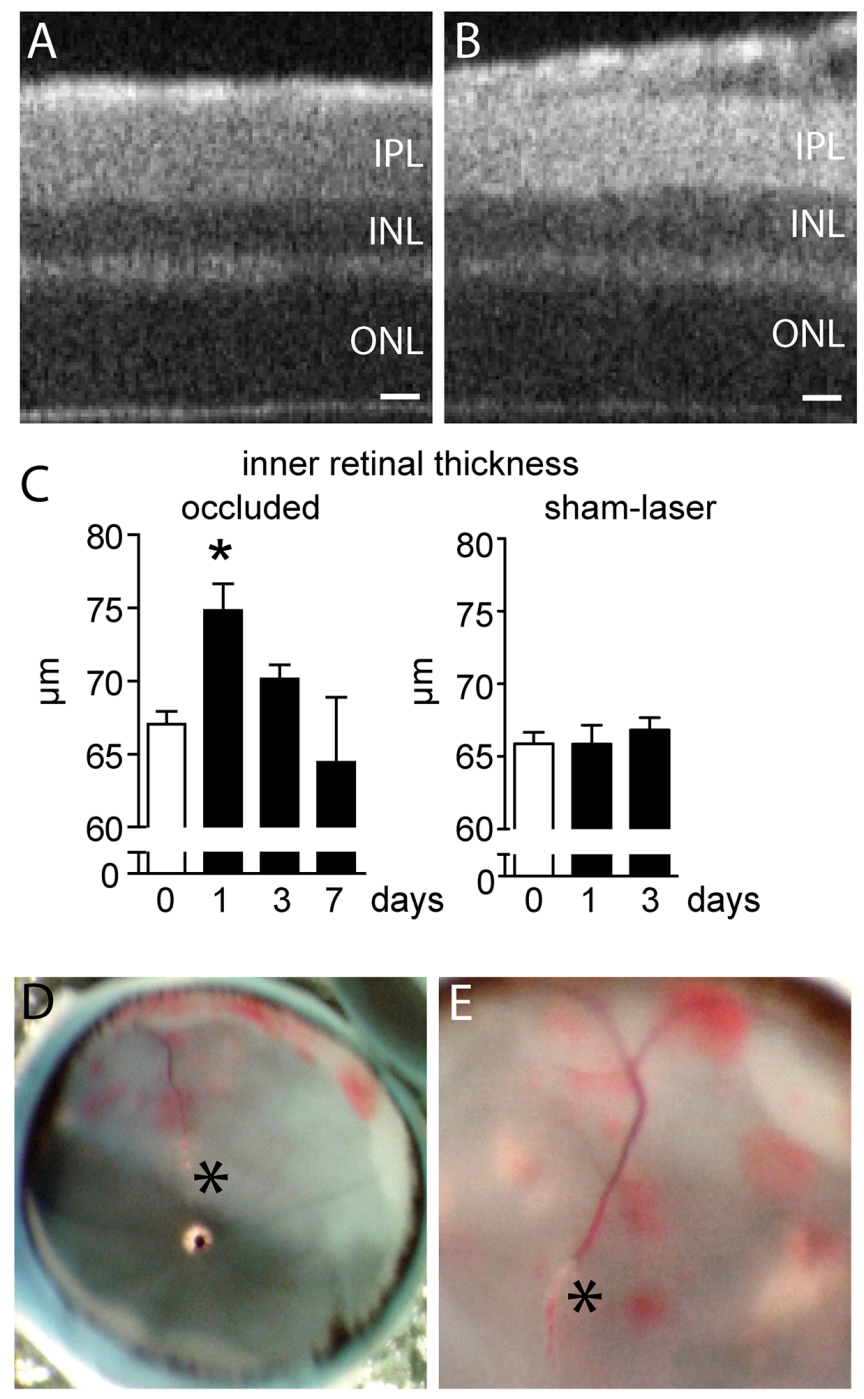
Retinal thickness and hemorrhages after experimental BRVO. (A and B) Representative SD-OCT image of the superior pole at ∼900μm of the optic nerve of a control retina (A) and after BRVO (B; ∼400μm upstream of the occlusion) at d1. (C) Quantification of the thickness of occluded and sham-lasered inner retina, measured from the OPL to the retina, at ∼900μm of the optic nerve at different time points. Values in histograms are mean ± SEM of thickness of inner retina from 3–16 eyes per group. Mann-Whitney non parametric test, * p<0.05 compared to control (0). Control are values of the retina before BRVO.D and E: Photographs of the appearance of inner retinal hemorrhages at d1 (* occlusion site). IPL: inner plexiform layer; INL: inner nuclear layer; ONL: outer nuclear layer; Scale bar A and B = 20μm.

Acute BRVO in patients presents characteristic clinical features with retinal edema and inner retinal hemorrhages [6]. To evaluate retinal changes after experimental BRVO in mice, we induced BRVO by direct laser coagulation to the superior vein at 2 to 3 disc diameter from the optic disc as we previously described [12]. The vein remained occluded in the majority of cases for the observation period of seven days [12] and eyes in which recanalization occurred were discarded from subsequent analysis. We first evaluated the retinas clinically by SD-OCT at 1, 3, and 7 days after the laser-induced occlusion. SD-OCT at ∼900μm distance from the optic nerve in non-occluded eyes (Fig 1A) and in the non-occluded inferior part of experimental eyes showed normal retinal layering (data not shown). In contrast, the inner retina (measured from the outer plexiform layer (OPL) to the vitreous) was thickened, at ∼900μm from the optic nerve, in the superior quadrant upstream of the occluded vein, 1 day after the occlusion (Fig 1B). Quantification of inner retinal thickness at d1, d3 and d7 revealed a transient significant thickening of the inner retina at d1 that resolved to normal levels by d3 (Fig 1C). Sham laser procedures, where the same amount of energy was applied at the same distance of the optic nerve, but in between two major vessels, without producing a vein occlusion, did not significantly increase the inner retinal thickness at ∼900μm (Fig 1C). During dissection of d1 eyes, the blood filled occluded vein and inner retinal hemorrhages of the upstream vascular bed was regularly observed (Fig 1D and 1E).

Taken together, our data shows that experimental BRVO in mice causes a transient inner retinal edema that coincides with increased vascular permeability, evidenced by the thickened inner retina and retinal hemorrhages respectively.

### Vascular Endothelial cells undergo apoptosis, followed by proliferation after BRVO

**Fig 2:**
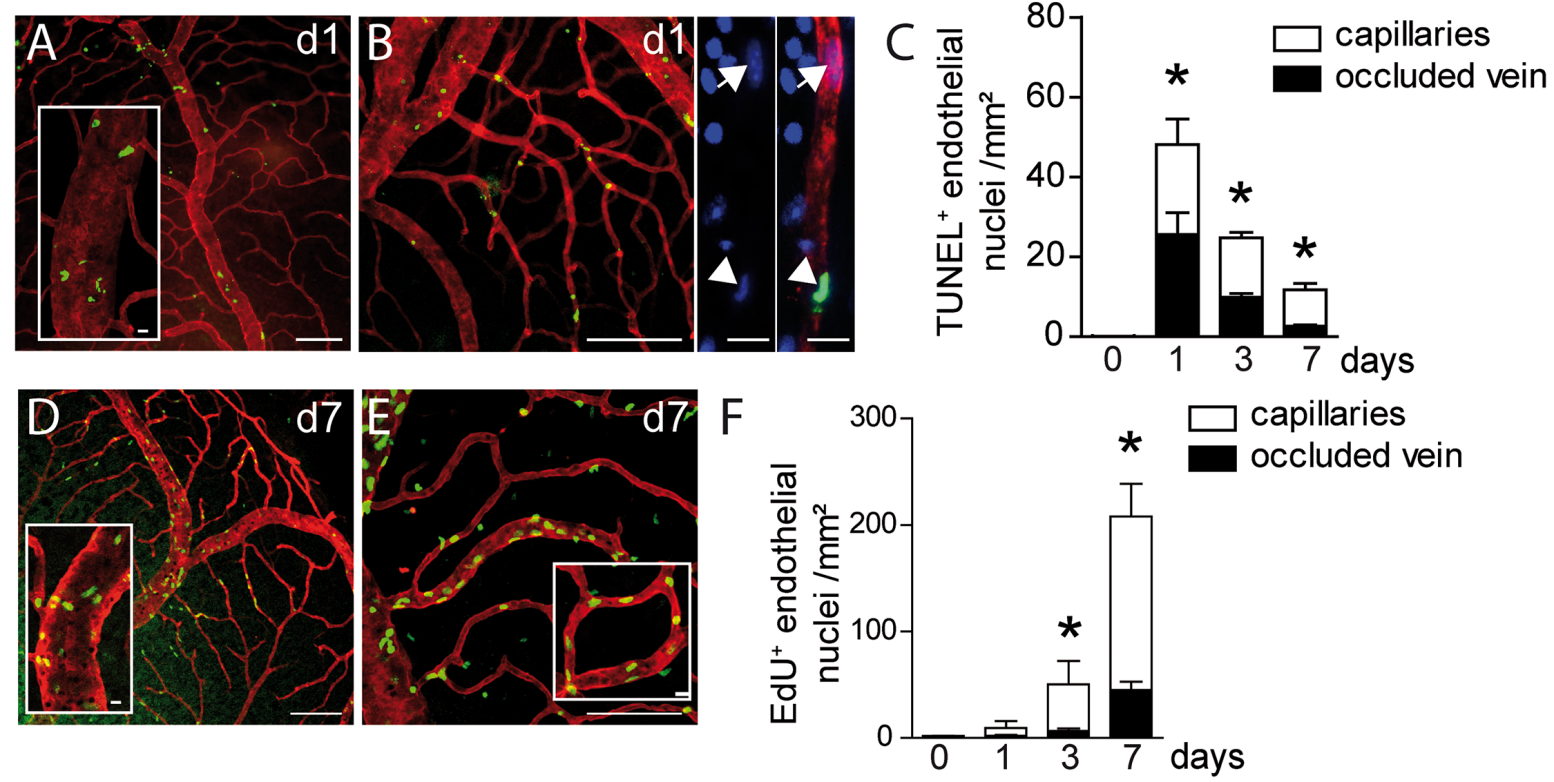
Vascular endothelial cell apoptosis and proliferation after BRVO. (A and B) Representative micrographs of TUNEL (green)—CollIV (red) double labeled retinal flatmounts at 1d after BRVO of the occluded vein (A) and the upstream capillary bed (B). (B- right panels) Detail of a DAPI(blue)-labelled upstream capillary depicting a normal nucleus (arrow) and an apoptotic TUNEL^+^nucleus (arrowhead). (C) Quantification of TUNEL^+^ECs of control retinas and in the occluded vein and upstream capillary bed at 1, 3 and 7d after BRVO. Values in histograms are mean ± SEM of number of TUNEL^+^ECs of retina from 5–10 eyes per group. Mann-Whitney non parametric test, * p<0.05 compared to control (0), intact retina or opposite part of the BRVO retina. (D and E) Representative micrographs of EdU (green)—CollIV (red) double labeled retinal flatmounts at 7d after BRVO of the occluded vein (D) and the upstream capillary bed (E). (F) Quantification of EdU positive ECs of control retinas and in the occluded vein and upstream capillary bed at 1, 3 and 7d after BRVO. Mice were daily injected intraperitoneally with EdU at d0 just after laser photocoagulation until sacrifice.Values in histograms are mean ± SEM of number of EdU+ cells of retina from 5–10 eyes per group. Mann-Whitney non parametric test, * p<0.05 compared to control (0), intact retina. Scale bars A, B, D, and E = 100μm, 20μm for insets, B right panels: 10μm.

Increased vascular permeability, which can lead to vasogenic edema, can be due to the overexpression of vasoactive substances, but also to an altered integrity of the vascular wall [16]. We had noticed in TUNEL-stained retinal flatmounts after BRVO that a significant number of TUNEL^+^cells seemed to associate with vascular structures. The laser coagulation site also invariably contained TUNEL^+^cells that were not considered in this analysis. To analyze apoptosis in the vascular compartment of the experimental retina we performed TUNEL (green staining)- Collagen IV (CollIV, red staining) double-labeling on retinal flatmounts of control and BRVO retina (Fig 2A). CollIV is strongly expressed by ECs and is a main component of the EC basal membrane. Staining with CollIV, endothelial specific markers lectin Bandeiraea simplicifolia, or CD102 revealed no apparent differences on BRVO retinal flatmounts. While only scarce TUNEL labeling was observed in the unaffected quadrant and in control eyes, we invariably observed a TUNEL^+^cells in the inner retina of the entire quadrant of the occluded vein up to its periphery. The quadrants of non-occluded veins were not affected. Surprisingly, 62% of TUNEL^+^nuclei in the BRVO retinas were ECs at d1. TUNEL^+^ECs were observed on the occluded vein (Fig 2A) and dependent capillaries (Fig 2B), identified by the CollIV co-staining. The mice were perfused with PBS prior to the staining and observation under the microscope clearly identified the TUNEL^+^cells as mural cells rather than intraluminal leucocytes. Quantification of TUNEL^+^ECs revealed a significant induction of apoptosis in both microvascular and macrovascular ECs at d1 that decreased towards d7, but stayed significantly elevated compared to controls (Fig 2C). At d7 TUNEL^+^ECs were mainly located in the capillaries.

Intrigued by the observation that ECs undergo apoptosis after BRVO, we next examined cell proliferation in mice that received daily injections of the traceable nucleotide analog EdU. To identify proliferating ECs we performed EdU (green staining)- CollIV (red staining) double-labeling on retinal flatmounts of control and BRVO retina. Control retinas contained rare EdU^+^cells in peripheral capillaries, but BRVO retinas contained numerous EdU^+^ECs in the occluded vein (Fig 2D) and upstream capillaries (Fig 2E) at d7. EdU^+^ECs in the non-occluded areas were scarce, suggesting BRVO induces only a minor vascular remodeling in the capillaries of the non-occluded vascular beds (not shown). Quantification of EdU^+^ECs after different time points of BRVO revealed a significant increase in their number at d3 and a further increase by d7 (Fig 2F). Interestingly, the majority of proliferating ECs were located in the capillaries upstream of the occluded vein. Nearly all EdU^+^cells were CollIV^+^, indicating that the vast majority of proliferating cells were ECs in BRVO. As EdU marks proliferating cells in a definite fashion the quantification reflects the number of ECs that proliferate since the occlusion and not the proliferative activity at the indicated day.

In summary, experimental BRVO leads to a wave of EC apoptosis that occurs concomitantly with the retinal edema. Subsequently, the EC proliferation rate increases, which is associated with a normalization of the retinal thickness. However, 7 days after the BRVO the turnover of ECs was still significantly increased, as EC apoptosis and proliferation was observed.

### The upstream capillaries dilate, but do not increase in density after BRVO

**Fig 3:**
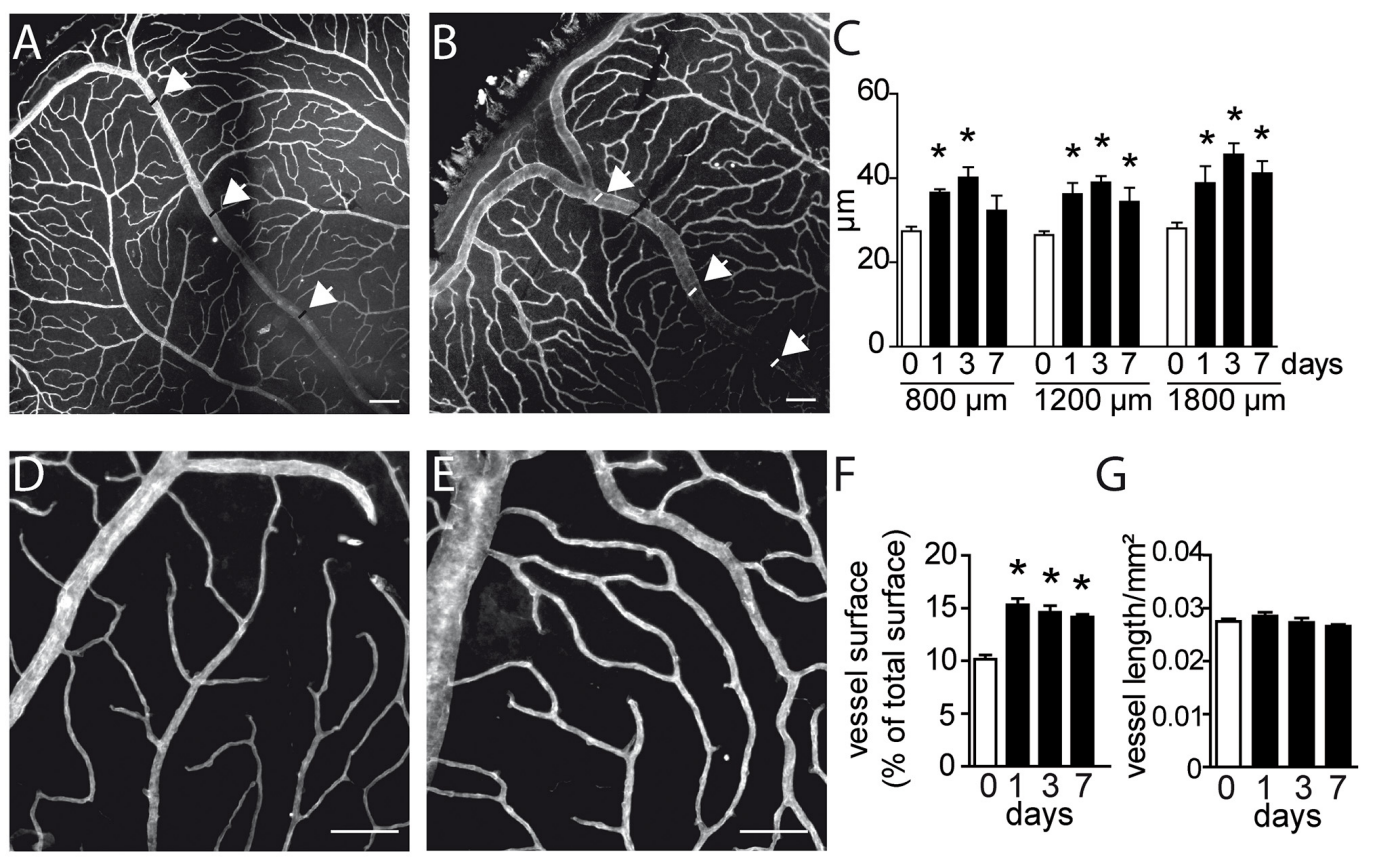
Morphometric analysis of vascular network remodeling after BRVO. (A and B) Representative micrographs of CollIV-labeled retinal flatmounts of a control retina (A) and of the occluded vein after BRVO at 3d (B). Positions of the measurements at 800, 1200 and 1600μm from optic nerve were indicated by arrowhead. (C) Measurements of the retinal vein diameter of control retinas or of non occluded side and in the occluded vein at 1, 3 and 7d and indicated distance from the optic nerve after BRVO. Values in histograms are mean ± SEM of diameter (μm) of control or occluded vein in the retina from 4–11 eyes per group. Mann-Whitney non parametric test, * p<0.05 compared to control (0), non occluded veins of the same eye and intact eyes. (D and E) Representative micrographs of CollIV-labeled retinal flatmounts of the superficial capillary network of a control retina (D) and upstream of the occluded vein at 3d (E). (F and G) Quantification of the surface covered by CollIV-positive capillaries (F) and length (capillary length per surface) at different time points. Values in histograms are mean ± SEM of surface or length of capillaries in the retina from 4–18 eyes per group. Mann-Whitney non parametric test, * p<0.001 compared to control (0), non occluded veins of the same eye and non-occluded eyes. Scale bar = 100μm.

To morphologically characterize the microvascular remodeling after experimental BRVO in mice, we next analyzed vessel parameters on CollIV-stained flatmounts of control (Fig 3A and 3D) and experimental (Fig 3B and 3E) mice. Immediately after BRVO one clinically observes the dilation of the upstream venous segment at the slit lamp. On CollIV stained flatmounts, the occluded vein (Fig 3B) appeared invariably wider compared to non-occluded veins of the same eye and intact eyes (Fig 3A) at 3d. As expected, measurements of the vein diameter, at increasing distances from the optic nerve and at different time points after the occlusion confirmed a significant increase in diameter (Fig 3C), which peaked at d3 but remained significantly elevated at d7. Similarly, the superficial capillaries upstream of the occluded vein appeared wider in experimental eyes (Fig 3E) compared to control eyes (Fig 3D). Quantification of the surface covered by CollIV^+^capillaries at different time points, by a previously described method [15], shows that the area covered by CollIV^+^capillaries increased significantly as soon as 1d after the occlusion and remained increased throughout the observation period (Fig 3F). The vessel density, measured as a function of capillary length per surface, remained however stable (Fig 3G). The increased capillary surface in conjunction with unchanged capillary length reflects capillary swelling in the absence of new capillary formation.

Taken together, our morphometric analysis shows a prolonged swelling with acute onset of the occluded vein and its upstream capillaries.

### BRVO leads to a significant loss of NG2^+^ pericytes

**Fig 4:**
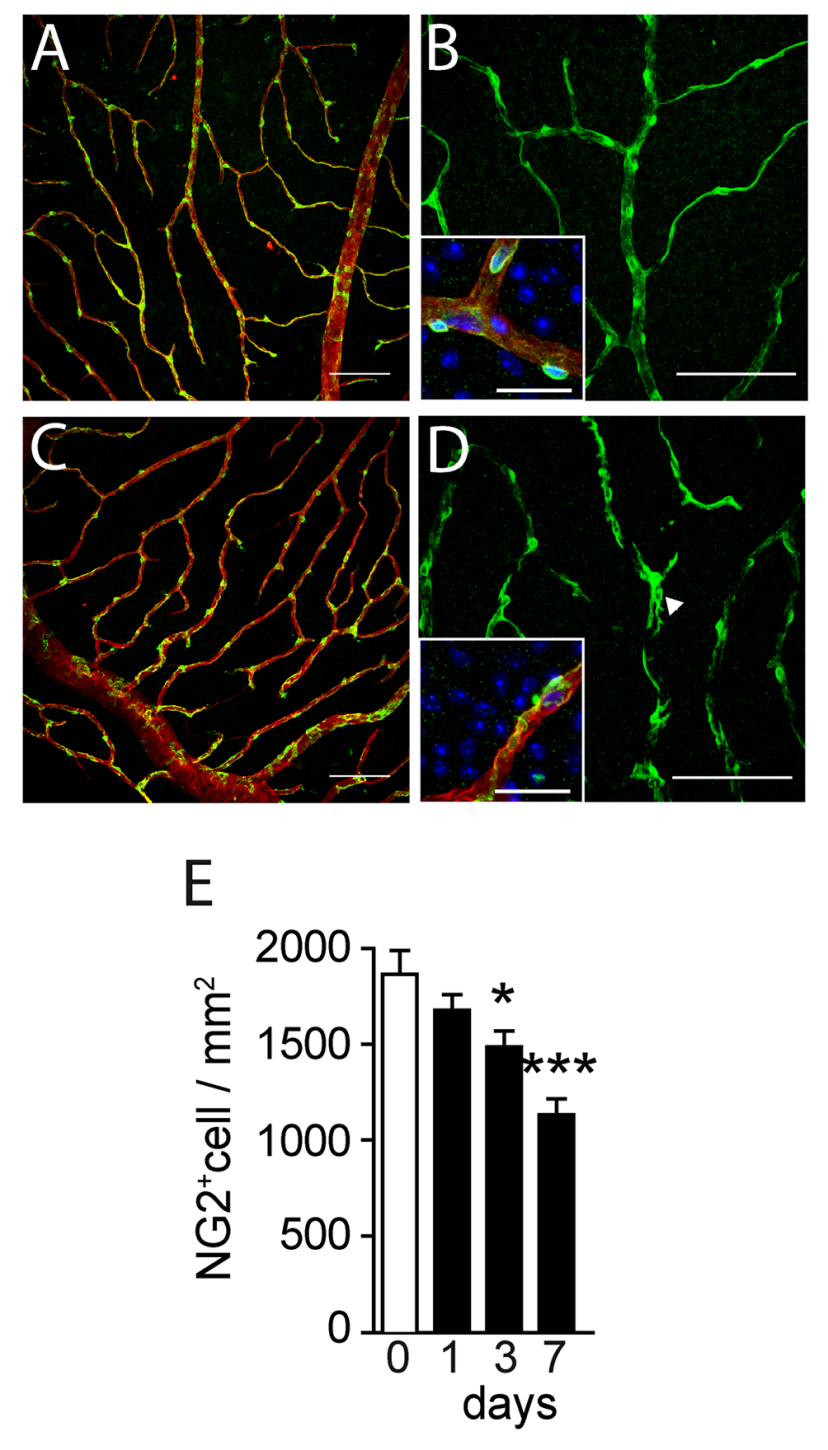
NG2-staining and pericyte counts after BRVO. (A-D) Representative micrographs of NG2 (green)-CollIV (red) double labeled retinal flatmounts of control (A and B) and at 7d after BRVO (C and D). DAPI is represented in blue in the insets of panel B and D. (E) Quantification of NG2 positive pericytes of control capillaries and capillaries upstream of the occluded vein at indicated time points. Values in histograms are mean ± SEM of number of NG2+ nuclei/mm² of vascular area. n = 4–18 per group. Mann-Whitney non parametric test, * p<0.05 compared to control (0), intact retina or opposite part of the BRVO retina. Scale bar = 100μm, 25 μm for insets.

Capillaries consist of endothelial cells surrounded by contractile pericytes (PC) that modulate capillary caliber, blood flow and stabilize ECs [17]. PCs are composed of a juxta-capillary cell body and multiple finger-like cytoplasmic processes that cover the abluminal side of capillaries. To analyze whether the PC coverage of the capillaries is altered in experimental BRVO we analyzed CollIV-stained flatmounts of control (Fig 4A and 4B) and experimental (Fig 4C and 4D) mice that were co-labeled with nerve/glial antigen 2 (NG2), a widely used PC marker in the retina. Contrary to trypsin digest preparations, this technique allows the evaluation of NG2+ pericytes within the context of the retinal tissue and is more accurate for the determination of pericyte densities in our experience. NG2 staining of control retina shows a strong signal of the cell body around PC nuclei (Fig 4B inset) and reveals the thin elongated processes of the PCs that cover the capillaries (Fig 4A and 4B). In contrast, PCs of the upstream capillaries 7d after BRVO featured shortened and thickened NG2^+^elongations and the CollIV^+^capillaries were less regularly covered as a result (Fig 4B and 4C). The NG2 staining of the PC perikarya appeared mottled and less regular (Fig 4D inset). Quantification of PCs at different time points after BRVO revealed a significant decrease three days after BRVO (-20.4%, Fig 4E). The contraction of the NG2^+^ pericyte population further progressed by d7, when 40% of the pericytes had been lost compared to intact and non-occluded capillary beds (Fig 4E). Interestingly, TUNEL^+^NG2^+^nuclei were only very rarely observed on TUNEL-NG2 double labeled retinas (data not shown), suggesting that PC dropout results from non-apoptotic cell death, dedifferentiation or migration as described in other pathologies [18–20].

These results reveal a severe loss of NG2^+^ PC coverage of the upstream capillary bed in BRVO.

### BRVO leads to the upregulation of pro-apoptotic and pro-proliferative mediators

**Fig 5:**
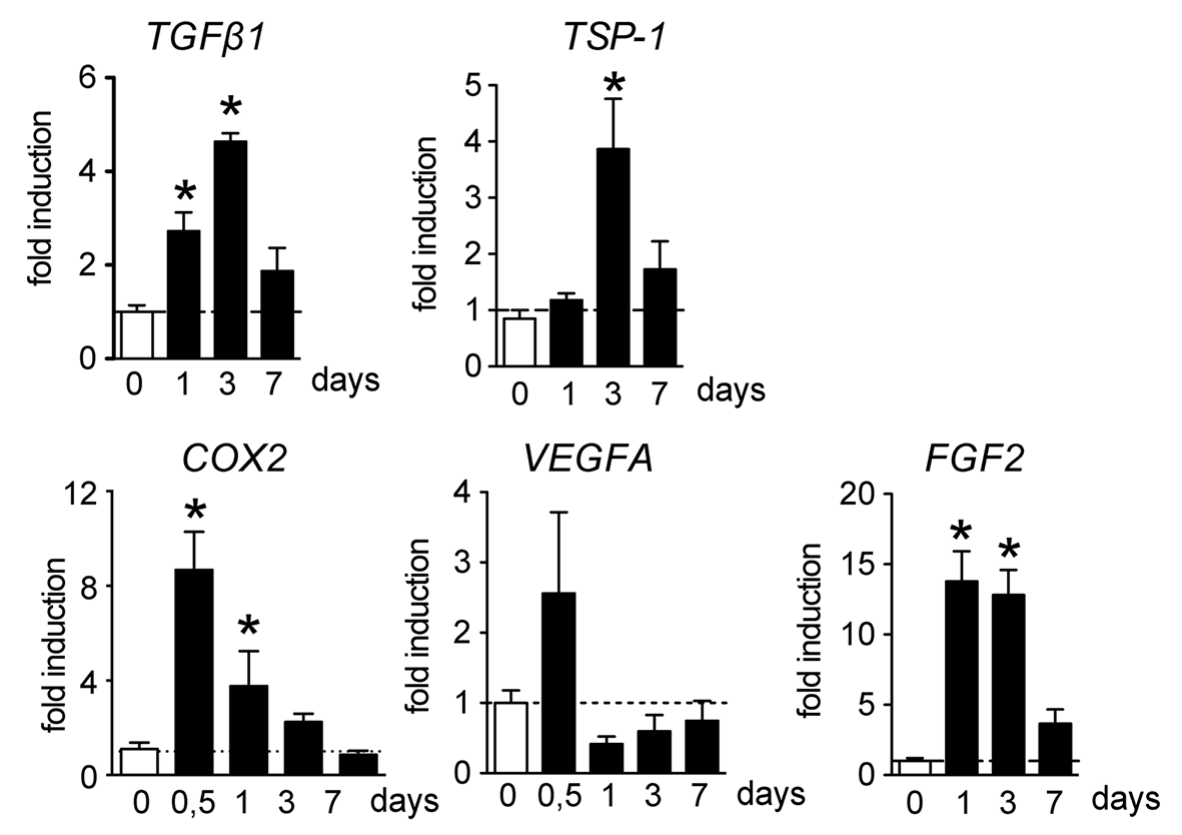
BRVO induced vascular and inflammatory markers expression. *TGFβ1*, *TSP-1*, *COX-2*, *VEGF*, and *FGF2* real-time RT-PCRs in the occluded retina at indicated time points. The results were normalized to S26 expression. Values in histograms are mean ± SEM of mRNA expression of occluded area from 4 eyes per group. Mann-Whitney non-parametric test, * p<0.05 compared to non occluded control.

TGFβ1 has been shown to induce angiogenesis *in vivo*, but its pro-angiogenic effect requires a transient proapoptotic step on ECs [21], somewhat similar to what we observe in BRVO. Indeed, real-time PCR of mRNA from the occluded section at different time points before (d0) and after BRVO reveals that *TGFβ1* mRNA expression is significantly increased at 1 and 3 days (Fig 5). *TSP-1* mRNA transcription, that participates in TGFβ1 activation but that can also induce endothelial apoptosis through other pathways [22], was equally significantly more transcribed 3d after the vein occlusion. Cyclooxygenase 2, that can lead to the production of vasoactive substances such as prostaglandin 2E [23] and thromboxane A2 [24], was significantly more transcribed after 12 and 24h after the occlusion and *VEGF* transcription had a tendency to increase at 12h. Interestingly, FGF-2, a potent inducer of EC proliferation [25], increased more than 10fold at d1 and d3 after BRVO (Fig 5).

Taken together, these results indicate that increased expression of several pro-apoptotic and pro-proliferative coincide with the observed endothelial cell remodeling.

### The weakened vascular network after BRVO becomes sensitive to hypoxia

**Fig 6:**
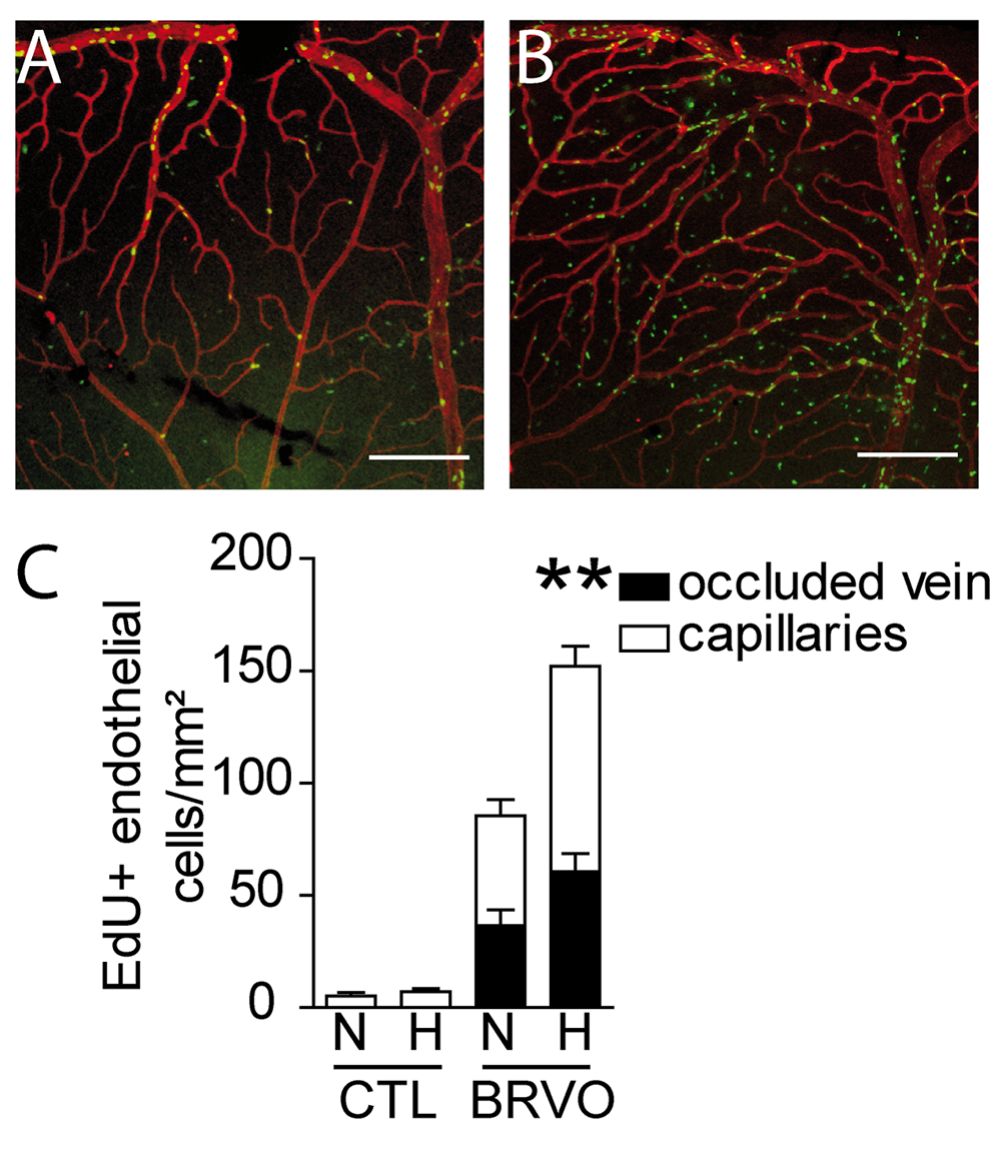
Hypoxia induced endothelial cell proliferation in control and BRVO retinas. (A and B) Representative micrographs of EdU (green)—CollIV (red) double-labeled retinal flatmounts at 7d after BRVO raised in normoxia (A) or hypoxia from d3 to d7 (B). All mice were daily injected with EdU from d3 to d7 before sacrifice (please not that retinas in Fig 2 were injected daily with EdU and are therefor not comparable in terms of numbers of EdU^+^cells). (C) Quantification of EdU positive ECs of the superior vein and upstream capillary bed at d7 of control retinas (without BRVO) and BRVO retinas, raised in normoxia and hypoxia. All mice were daily injected with EdU from d3 to d7 before sacrifice. Values in histograms are mean ± SEM of mRNA expression of occluded area from 9–10 retinas per group. Mann-Whitney non parametric test, ** p<0.01, normoxic BRVO versus hypoxic BRVO. Scale bars AB = 200μm.

PC dropout leads to a destabilization of the vascular network [17]. To test whether the loss of PCs we observe after BRVO leads to vascular destabilization, we exposed control and BRVO animals to hypoxia of 10% O_2_ (from d3 to d7) or normal atmosphere (20,9% O_2_) and evaluated EC proliferation using EdU incorporation at d7. EdU (green)- CollIV (red) double stained retinal flatmounts of 7d BRVO retina that were kept under normoxic conditions (Fig 6A) and exposed to hypoxia (Fig 6B) showed a dramatic increase in EdU^+^ECs in the hypoxic condition. Quantification of EdU^+^ECs showed that hypoxia did not significantly increase cell proliferation in non-occluded control retina (Fig 6C; 5.25 EdU^+^ECs /mm² for normoxia versus 7.25 EdU^+^ECs /mm² for hypoxia). In contrast, hypoxic conditions nearly doubled EC proliferation in the occluded retina with BRVO (Fig 6C; 85.46 EdU^+^ECs /mm² for normoxia versus 152.2 EdU^+^ECs /mm² for hypoxia). Please note that the values of Edu+cells in normoxic BRVO retina are lower in this set of experiments compared to the results presented in Fig 2, as EdU was only injected from d3 to d5 (d0 to d7 in Fig 2F).

In summary, our results show that the vascular network after BRVO becomes greatly more susceptible to hypoxic conditions.

## Discussion

We here investigated the acute and sub-acute vascular changes following experimental BRVO in mice. The most straightforward and not surprising observation after experimental BRVO is the acute (d1) and sustained (d7) swelling of the occluded vein and its upstream capillary bed that we here quantify. The acute phase of the occlusion (d1) revealed also an unexpected wave of EC apoptosis in the entire quadrant (veins and capillaries) upstream of the occluded vein. While EC cells were not the only cells undergoing apoptosis after BRVO, they constituted a solid 62% of TUNEL^+^cells at d1. The identity of the remaining apoptotic cell populations is currently under investigation. The apoptosis was not a direct consequence of the laser coagulation, apoptotic cells at the laser-coagulation site were excluded from the analysis, and they were distributed in the upstream vascular bed at greater distances from the injury. Central veins and capillaries of non-occluded vessels, even though there were closer to the impact, were not affected. The initial wave of EC apoptosis might be due in part to the increased expression of apoptotic signals such as TGFβ1 and TSP1, that we show to be temporarily increased after the occlusion. Indeed, TGFβ1 has been shown to transiently induce EC apoptosis, before participating in angiogenesis [21], somewhat similar to what we observe in BRVO. We have previously shown that TSP-1 induction in oxygen-induced retinopathy participates EC apoptosis via CD36 [26] and a similar mechanism might participate in EC apoptosis in BRVO. However our study only shows an association of increased expression of these factors with EC apoptosis and functional studies are needed to evaluate their actual role in the phenomenon.

Similar to the human disease and concomitantly with the vessel dilation and apoptosis we also observed an inner retinal thickening and hemorrhages that are likely the functional consequence of the EC apoptosis and increased secretion of vascular permeability factors, such as prostaglandins and VEGF, in this model at this early stage. Subsequently, ECs start to proliferate, which likely participated in the improved function of the blood-retinal barrier, as the retinal thickening subsided. Interestingly, while VEGF transcription did not increase dramatically in the BRVO model in our hands, we observed a very significant increase in FGF2 that might partake in the EC proliferation. However, the EC turnover remained pathologically high, as both, EC apoptosis and proliferation remained well above the levels in controls even 7 days after the occlusion, when FGF2-transcription had returned to near normal levels.

Interestingly we also observe a significant dropout of NG2^+^ PCs starting at d3. By d7 40% of the initial PC population was lost in the section of the occluded vein. PCs physiologically modulate capillary caliber, blood flow and stabilize ECs [17]. Their degeneration might therefore further destabilize the ECs and participates in the prolonged capillary dilation and EC turnover we observe at d7. Interestingly, TUNEL^+^ECs at d7 were mainly found in the capillaries, which might reflect the lost stabilizing effect due to the PC degeneration. In longer terms, this early PC dropout might pave the way for capillary loss and subsequent chronic ischemia and edema that characterize the late stage disease.

Finally, we show that the vascular network upstream of the occlusion has become functionally destabilized, as exposure of the experimental animals to hypoxia from d3 to d7, when PCs dropout induces a dramatic two-fold increase in EC proliferation.

Our results from experimental BRVO in mice therefore suggest that the stagnation of blood flow is associated with a wave of EC death that concurs with inner retinal edema and hemorrhages. Subsequently, the blood-retinal barrier reforms, while ECs proliferate. However, the vascular network upstream of the occlusion remains destabilized, characterized by a pronounced NG2^+^ PC dropout and an un-physiologically high EC turnover.

To our knowledge, our study is the first to describe EC apoptosis, EC proliferation, and PC dropout associated with experimental BRVO in the mouse. Experimental BRVO in mice is likely different from the human disease due to the naturally occurring peripheral vein collateral. Nonetheless, PC loss was demonstrated in experimental BRVO in the monkey a long time ago [27] suggesting that similar mechanisms take place in primates and therefore possibly in the human disease.
